# The Multi-Modal Effect of the Anti-fibrotic Drug Pirfenidone on NSCLC

**DOI:** 10.3389/fonc.2019.01550

**Published:** 2020-01-21

**Authors:** Sebastian Marwitz, Kati Turkowski, Dörte Nitschkowski, Andreas Weigert, Julius Brandenburg, Norbert Reiling, Michael Thomas, Martin Reck, Daniel Drömann, Werner Seeger, Klaus F. Rabe, Rajkumar Savai, Torsten Goldmann

**Affiliations:** ^1^Pathology, Research Center Borstel – Leibniz Lung Center, Borstel, Germany; ^2^Airway Research Center North (ARCN), Member of the German Center for Lung Research (DZL), Borstel, Germany; ^3^Molecular Mechanisms in Lung Cancer, Member of the German Center for Lung Research (DZL), Member of the Cardio-Pulmonary Institute (CPI), Bad Nauheim, Germany; ^4^Department of Internal Medicine, Member of the DZL, Member of CPI, Justus Liebig University, Giessen, Germany; ^5^Faculty of Medicine, Institute of Biochemistry I, Goethe-University Frankfurt, Frankfurt, Germany; ^6^Microbial Interface Biology, Research Center Borstel – Leibniz Lung Center, Borstel, Germany; ^7^Department of Thoracic Oncology, University Hospital Heidelberg, Heidelberg, Germany; ^8^Translational Lung Research Center Heidelberg, Member of the German Center for Lung Research (DZL), Heidelberg, Germany; ^9^Department of Thoracic Oncology, LungenClinic Grosshansdorf, Großhansdorf, Germany; ^10^Medical Clinic III, University Medical Center Schleswig-Holstein (UKSH), Lübeck, Germany; ^11^Department of Pneumology, LungenClinic Grosshansdorf, Großhansdorf, Germany; ^12^Frankfurt Cancer Institute, Goethe University, Frankfurt am Main, Germany

**Keywords:** TGFβ, lung cancer, immunotherapy, tumor microenvironment, SMAD

## Abstract

Although immune checkpoint and targeted therapies offer remarkable benefits for lung cancer treatment, some patients do not qualify for these regimens or do not exhibit consistent benefit. Provided that lung cancer appears to be driven by transforming growth factor beta signaling, we investigated the single drug potency of Pirfenidone, an approved drug for the treatment of lung fibrosis. Five human lung cancer cell lines and one murine line were investigated for transforming growth factor beta inhibition via Pirfenidone by using flow cytometry, In-Cell western analysis, proliferation assays as well as comprehensive analyses of the transcriptome with subsequent bioinformatics analysis. Overall, Pirfenidone induced cell cycle arrest, down-regulated SMAD expression and reduced proliferation in lung cancer. Furthermore, cell stress pathways and pro-apoptotic signaling may be mediated by reduced expression of Survivin. A murine subcutaneous model was used to assess the *in vivo* drug efficacy of Pirfenidone and showed reduced tumor growth and increased infiltration of T cells and NK cells. This data warrant further clinical evaluation of Pirfenidone with advanced non-small cell lung cancer. The observed *in vitro* and *in vivo* effects point to a substantial benefit for using Pirfenidone to reactivate the local immune response and possible application in conjunction with current immunotherapies.

## Introduction

Lung cancer is the leading cause of cancer-related death worldwide with an estimated 1.6 million of deaths per year. Non-small cell lung cancer (NSCLC) accounts for 80–85% of diagnosed lung cancer cases (1) and the majority of patients are diagnosed with metastatic disease in the absence of specific early symptoms (2). The implementation of targeted therapies for patients with treatable oncogenic alterations and immunotherapies, either alone or in combination with chemotherapy, has substantially improved the prognosis of patients with advanced, unresectable NSCLC (3) and changed the treatment paradigm with the introduction of anti-PD-1 treatment as first line therapy (4). Despite the fact that immune checkpoint therapies offer remarkable responses, many NSCLC patients will not qualify for either targeted therapies or immune checkpoint inhibitors. Hence, novel therapeutic targets are required to provide these patients enhanced treatment options other than palliative care. The transforming growth factor β (TGFβ) pathway has recently been shown to exhibit aberrant activation in human NSCLC patient tissues, mediated by epigenetic silencing of the pseudo-receptor BMP and activin membrane-bound inhibitor (BAMBI) that resulted in increased Mothers against decapentaplegic homolog 3 (SMAD3) phosphorylation, tumor growth, and Epithelial-Mesenchymal-Transition (EMT) signatures (5). Furthermore, EMT signatures in human NSCLC specimen were inversely associated with T cell infiltration and exhibited increased expression of immune checkpoint molecules (6). Simultaneous inhibition of PD-L1 and TGFβ, by using a bifunctional fusion protein, effectively reverted the mesenchymalization and chemotherapy resistance of human lung cancer cells (7), which highlights the possible benefit of addressing both, the tumor as well as infiltrating immune cells. Recent approaches aiming at reprogramming of the tumor-microenvironment (TME) by either targeting cancer-associated fibroblast mediated immune evasion (8) or TGFβ signaling, may enhance (immune)-therapy (9), improve vaccine approaches (10) or chemotherapy (11). Nevertheless, these promising approaches still remain in pre-clinical phase. A retrospective study in idiopathic pulmonary fibrosis (IPF), which is discussed to be driven by aberrant TGFβ (12), found that patients treated with Pirfenidone had a significantly reduced risk of developing lung cancer during their follow-up phase (13). Pirfenidone is a FDA/EMA approved drug for treating IPF (14) that inhibits TGFβ1 induced expression of Collagen type I and Heat Shock Protein 47 (HSP47) in human lung adenocarcinoma cells (15) and was recently shown to suppress proliferation in human pancreatic cancer cells (16) or inhibit motility of NSCLC cells by interfering with the urokinase system (17).

The present study therefore aimed to conduct *in vitro* and *in vivo* investigations of the single-agent potency of Pirfenidone for treating lung cancer and exploring the rationale of possible application in NSCLC patients not eligible for chemotherapy or immune checkpoint therapy by off-label use.

## Materials and Methods

### Cell Culture

Human adenocarcinoma (A549, H838, H1650, H1975), squamous cell carcinoma (H520) and mouse Lewis lung carcinoma 1 cells (LLC1) were purchased from the American Type Culture Collection (ATCC, Manassas, VA) and maintained in RPMI-1640 supplemented with 10% fetal calf serum (FCS, Invitrogen, Carlsbad, CA, USA or Pan Biotech, Aidenbach, Germany) and 1%P/S (Invitrogen or Pan Biotech) and 1% L-Glutamine (Invitrogen or Pan Biotech). Cells were incubated at 37°C in 5% CO_2_. Parts of the cell culture experiments were conducted in two independent laboratories and by two independent observers (SM, KT) and are indicated as such.

### Cell Line Authentication

Human cell line authentication via STR analysis was done in a DIN ISO 17025 certified service laboratory (Eurofins Genomics, Ebersberg, Germany). Results were submitted to online STR profile search at DSMZ (Braunschweig, Germany) and evaluation values retrieved from interrogating 9 STRs: A549 (1.0; 36/36), H1650 (1.0; 36/36), H838 (0.89; 32/36), H1975 (1.0; 36/36), H520 (0.89; 32/36). All cell lines used were tested for mycoplasma and were free of mycoplasma at time of experiments.

### Reagents

All experiments used Pirfenidone (CAS RN: 53179-13-8) from the same vendor (TCI Deutschland GmbH, Eschborn, Germany), reconstituted in pre-warmed PBS for *in vitro* experiments or methylcellulose for *in vivo* experiments.

### Cell Cycle Analysis

To perform cell cycle analysis by flow cytometry, the cells were seeded into T75 cell culture flasks and left over night to adhere. The next day, cell culture media was replaced by fresh media containing Pirfenidone or the respective amount of PBS as solvent control. Treatment was continued for 48 h in a CO_2_ incubator at 37°C. Cell culture media was removed to eliminate dead cells and the remaining cell layer was washed with PBS. The cells were detached by using trypsin/EDTA and washed with PBS. A total of 0.5 × 10^6^ cells were transferred to 5 ml flow cytometry tubes and fixed with final 1% PFA for 10 min at 4°C. A 1 ml aliquot of flow cytometry buffer (PBS with 1% heat inactivated FCS and 0.09% sodium azide; sterile-filtrated) was added and the cells were pelleted at 300 x g for 5 min and the supernatant discarded. Next, cells were permeabilized with 0.25% Triton X-100/PBS for 7 min at RT. Two milliliters of flow cytometry wash buffer was added and the cells were pelleted by centrifugation for 5 min at 300 x g to discard the supernatant. A 500 μl aliquot of 3 μM DAPI/PBS solution (Biolegend, San Diego, CA, USA) was used to resuspend the cells and stain intranuclear DNA for 15 min at RT in the dark. The cells were analyzed on a Macs Quant analyzer (Miltenyi, Bergisch-Gladbach, Germany) with DAPI detectors set to linear range. Cell cycle analysis was performed using FCS Express software version 6 (DeNovo Software, Glendale, CA, USA) and the proprietary multi-cycle analysis tool. For this, cellular debris was excluded by gating on a homogenous population and the gates were set via FSC vs. SSC to focus on single cells. Furthermore, additional gating was performed via DAPI-H vs. DAPI-W to focus on single cells for multi cycle analysis.

### In-Cell Western

An In-cell Western (ICW) assay was used to detect and quantify relative abundance of proteins. 5 × 10^3^ cells in 100 μl of media per well were seeded on a 96-well plate and left to adhere overnight. On the next day, the cell culture media was changed to treat the cells with either Pirfenidone, or a respective volume of PBS or left untreated for the indicated time-points. After treatment, the cells were fixed with a final 2% PFA in the cell culture medium for 10 min before the supernatant was discarded. The cells were permeabilized with 50 μl of 0.25% Triton X-100/PBS for 7 min at RT and the volume removed afterwards. The cells were then washed 3x with 150 μl of PBS and blocked finally with 100 μl 1x Roti-Block (Carl Roth, Karlsruhe, Germany) for 1.5 h at RT. Primary antibodies were diluted in 50 μl 1x Roti-Block and left for incubation for 1 h at RT under constant gentle shaking. Negative controls with omission of primary antibody were included for each experimental condition and on every plate. Supernatant was removed and the wells were washed 3x with 150 μl of PBS. Primary antibodies were detected using either goat anti-mouse or goat anti-rabbit IgG conjugated with IRDye® 800CW (Licor Biosciences, Lincoln, NE, USA) at a dilution of 1/1,200 in 1x Roti-Block supplemented with TO-PRO3-Iodide (ThermoFisher Scientific, Waltham, MS, USA) for 1 h at RT. The wells for background controls did not receive TO-PRO3-Iodide to allow subtraction of pure 800 nm background signal. After incubation with secondary antibodies, the supernatant was removed and the wells washed 3x with 150 μl PBS. Plates without liquids were scanned on an Odyssey Clx near-infrared scanner (Licor Biosciences) using “auto scan” on 800 and 700 nm channel as intensity settings and a 3 mm focus off-set at 169 μm resolution with lowest quality. For semi-quantitative analysis of protein expression, the ImageStudio software version 4 (Licor Biosciences) was used. The shift of images between the two channels was corrected prior to analysis within ImageStudio and a grid was placed on the wells to measure the relative fluorescence signal. The mean background signal was calculated and subtracted from each experimental parameter. The TO-PRO3-Iodide signal was used to normalize target expression to the respective cell number each well and the mean signal from 3 technical replicates was calculated for each experiment.

### Antibody Arrays

Antibody arrays were used to analyze whole cell lysates from the cell culture experiments. The PathScan Intracellular Signaling and the PathScan Stress and Apoptosis arrays (Cell Signaling Technology, Danvers, MA, USA) were used according to manufacturer's instructions. In short, adherent cells from stimulation experiments were detached using Trypsin/EDTA, washed with PBS and transferred to a microcentrifuge tube. Then, 1x Cell Lysis Buffer (Cell Signaling Technology) was added according to manufacturer's instructions and protein concentration was determined using BCA test. 0.75 mg/ml of total protein was used to incubate on the array slide and later on incubated with a biotinylated secondary antibody. Bound proteins were detected by addition of streptavidin-conjugated DyLight® 680 and the fluorescence image was acquired on an Odyssey Clx near-infrared scanner (Licor Biosciences). For detection the 700 nm channel was used and the intensity was set auto exposure with a resolution of 21 μm and medium image quality with no focus off-set. Image analysis done using ImageStudio software version 4.0 and a grid was place on the scanned image. Then the intensity values were exported to spreadsheet software. Signal from negative control spots of each array was substracted from raw expression signals of targets present on each array. For each target 2 technical replicates were present on the antibody arrays and the median of both values was used for further analysis.

### Immunohistochemistry (IHC)

IHC was conducted as described elsewhere (5) with the following antibodies and dilutions: rabbit anti-CD4 (1/1,000, clone EPR19514, abcam, Cambridge, UK), rabbit anti-CD19 (1/800, clone D4V4B, Cell Signaling Technology, Danvers, MS, USA), rabbit anti-NKp46/NCR1 (1/500, polyclonal, NSJ Bioreagents, San Diego, CA, USA), rabbit anti-CD45 (1/250, clone EP322Y, abcam), rabbit anti-CD3 (1/100, polyclonal, abcam), rabbit anti-CD8 alpha (1/2,000, clone EPR21769, abcam).

### Transcriptome Analysis

Total RNA was used for reverse-transcription, amplification, and labeling using the Low-Input Quick Amp Labeling Kit (Agilent Technologies, Böblingen, Germany) according to manufacturer's instructions and described elsewhere (5). A Moderated *T*-Test with a fold-change cut-off of ≥2 as well as a Westfall-Young Permutation family-wise error rate (FWER) of ≤ 0.05 as multiple testing correction were applied to retrieve significantly regulated genes. The g:Profiler web-tool with a false discovery rate (FDR) cut-off of ≤ 0.05 was used to for enrichment of pathways. Molecular interactions were retrieved from String database with a confidence cut-off filter ≥0.7 allowing a maximum of 20 first shell interactors and 5 2nd shell interactors. Interaction data was loaded into Cytoscape software version 3.6.

### Real-Time Cell Analysis (RTCA)

To monitor the cellular behavior in real time, the xCELLigence RTCA SP (Acea Biosciences, San Diego, CA, USA) was used according to the manufacturer's instructions. For this, cells were seeded on an E-Plate 96-well plate and left to adhere prior to stimulation. The next day, the cell culture medium was changed and measurement was started with an initial measurement every 30 s for 5 h and then every 30 min for 75 h. The background signal of the well plate with medium containing no cells was subtracted. Every experimental parameter was run with 4 technical replicates and the mean of each was used for statistical analysis. Cellular impedance of mean values was displayed over time as the “normalized cell index” and normalization was performed with unstimulated control cells.

### Scratch Assay

Cells were seeded into 24-well plates and cultured with respective media RPMI-1640 until they reached a confluence of 100%. A vertical scratch through the plate was produced with a 10 μl pipette tip. After washing with PBS, the cells were incubated in RPMI-1640 containing 1.5 mg/ml Pirfenidone. Immediately after wounding and at 20 h, scratches were photographed at 5 x magnification with phase contrast (Leica DMI3000 B microscope, Leica, Germany). Extents of wounds were measured and calculated with ImageJ Software (NIH). Four technical replicates were conducted for each experiment (*N* = 3).

### Proliferation Assay

Proliferation assays were performed with serum-starved cells exposed for 24 h to RPMI-1640 supplemented with 1.5 mg/ml Pirfenidone using a BrdU colorimetric cell proliferation ELISA according to manufactures instructions (Roche, Basel, Switzerland). Absorbance was measured at 370 nm with reference at 492 nm in a plate reader (TECAN, Männedorf, Switzerland). The proliferation of cells was plotted as a percentage of absorbance compared with the control cells' absorbance. At least three independent experiments were performed with 8 technical replicates each.

### Migration Assay

Chemotactic migration was quantified by using a Boyden chamber transwell assay (8 μm pore size; uncoated filters; Corning, New York, NY, USA). Either control or Pirfenidone 1.5 mg/ml RPMI-1640 supplemented media was provided in the lower chamber and tumor cells in serum free RPMI-1640 were introduced to the upper chamber (5 × 10^4^). The cells were allowed to migrate for 16 h and then fixed with methanol and washed in PBS. Cells on the apical side of membranes were removed with cotton swaps. Remaining cells on the membrane were stained with crystal violet and imaged using a digital slide scanner (Nanozoomer 2.0 HT, Hamamatsu, Japan). ImageJ software (NIH, MD, USA) was used for image analyses. At least three independent experiments were performed with 4 technical replicates. Migration was plotted as percentage of Pirfenidone treated cells compared to PBS control.

### Mouse Model

All experiments using animal models were performed according to the German Law for Animal Protection and the National Institute of Health Guidelines for Care and Use of Laboratory Animals. The protocol for the animal studies was approved by the University Animal Care Committee and the federal authorities for animal research of the Regierungspräsidium Darmstadt (Hessen, Germany; approval number B2/1107). C57BL6/J mice were purchased from Charles River Laboratories (Sulzfeld, Germany). Lewis lung carcinoma cells (1 × 10^6^) were randomly injected subcutaneously into 6–8 week old BL6/J mice. Pirfenidone was applied in 2 dosages in methyl cellulose via oral gavage on a daily basis and sacrificed after 20 days. Control groups received methyl cellulose.

### Flow Cytometry

To prepare tumors for flow cytometric analysis of immune cell composition, the tumors were placed in 35 × 10 mm Petri dishes (Sarstedt, Germany) and cut into small cubes (<1 mm^3^) with a scalpel. A digestion solution (2 ml) of collagenase D (0.2 mg/ml; Roche Diagnostics), pronase (1 mg/ml; Roche Diagnostics, Basel, Switzerland), and 2 ml of deoxyribonuclease I (400 U/ml, Promega, Madison, W, USA) was added, and samples were incubated for 40 min at 37°C on a shaker. The digested tissue was resuspended in 10 ml of PBS with 10% FCS to stop the digestion reaction and centrifuged for 5 min at 1,600 rpm. The pellet was washed with PBS and passed through a 40-mm cell strainer (BD Biosciences), and cells were counted. The cell suspension was then centrifuged, resuspended in 1 ml ice-cold 1% paraformaldehyde per 106 cells, and fixed for 15 min on ice. After centrifugation, cells were resuspended in FACS buffer [PBS, 0.15% EDTA, 10% FCS (pH 7.2)] and stained with fluorochrome-conjugated antibodies and analyzed on a LSRII/Fortessa flow cytometer (BD Biosciences, Heidelberg, Germany). Data were analyzed using FlowJo software Vx (Treestar). Antibodies and secondary reagents were titrated to determine optimal concentrations. CompBeads (BD Biosciences, Heidelberg, Germany) were used for single-color compensation to create multi-color compensation matrices. For gating, fluorescence minus one (FMO) controls were used. The instrument calibration was controlled daily using Cytometer Setup and Tracking beads (BD). The following antibodies were used: anti-CD3-PE-CF594, anti-CD4-BV510, anti-CD8-BV650, anti-CD11c-AlexaFluor700, anti-CD19-APC-H7, anti-Ly6C-PerCP-Cy5.5, anti-NK1.1-PE (BD Biosciences), anti-CD45-VioBlue, anti-MHC-II-APC (Miltenyi Biotec, Bergisch-Gladbach, Germany), anti-CD11b-BV605, anti-F4/80-PE-Cy7, anti-GITR-FITC, and anti-Ly6G-APC-Cy7. 7-AAD was used for dead cell exclusion.

### Data and Statistical Analyses

Statistical significance was determined by using GraphPad Prism (GraphPad Software, San Diego, CA, US) and the two-tailed unpaired or paired (where applicable) Student's *t*-test as well as one-way or two-way analysis of variance (1W/2W ANOVA) as indicated in the figure legends. Comparison of two experimental groups with one control group was calculated using 1W ANOVA with Dunnett's multiple comparisons test. Data is shown as mean ± SD of independent experiments unless otherwise stated. *P*-values are abbreviated as following: with ^*^p ≤ 0.05, ^**^p ≤ 0.01, ^***^p ≤ 0.001, ^****^p ≤ 0.0001.

### Ethics Approval and Consent to Participate

All experiments using animal models were performed according to the German Law for Animal Protection and the National Institute of Health Guidelines for Care and Use of Laboratory Animals. The protocol for the animal studies was approved by the University Animal Care Committee and the federal authorities for animal research of the Regierungspräsidium Darmstadt (Hessen, Germany; approval number B2/1107).

### Availability of Data and Materials

The datasets used and/or analyzed during the current study are available from the corresponding author on reasonable request. Transcriptome data will be publicly available at the GEO database upon publication under GSE136935.

## Results

### Pirfenidone Induced Cell Cycle Arrest and Reduced Proliferation in NSCLC Cell Lines

**Figure 1:**
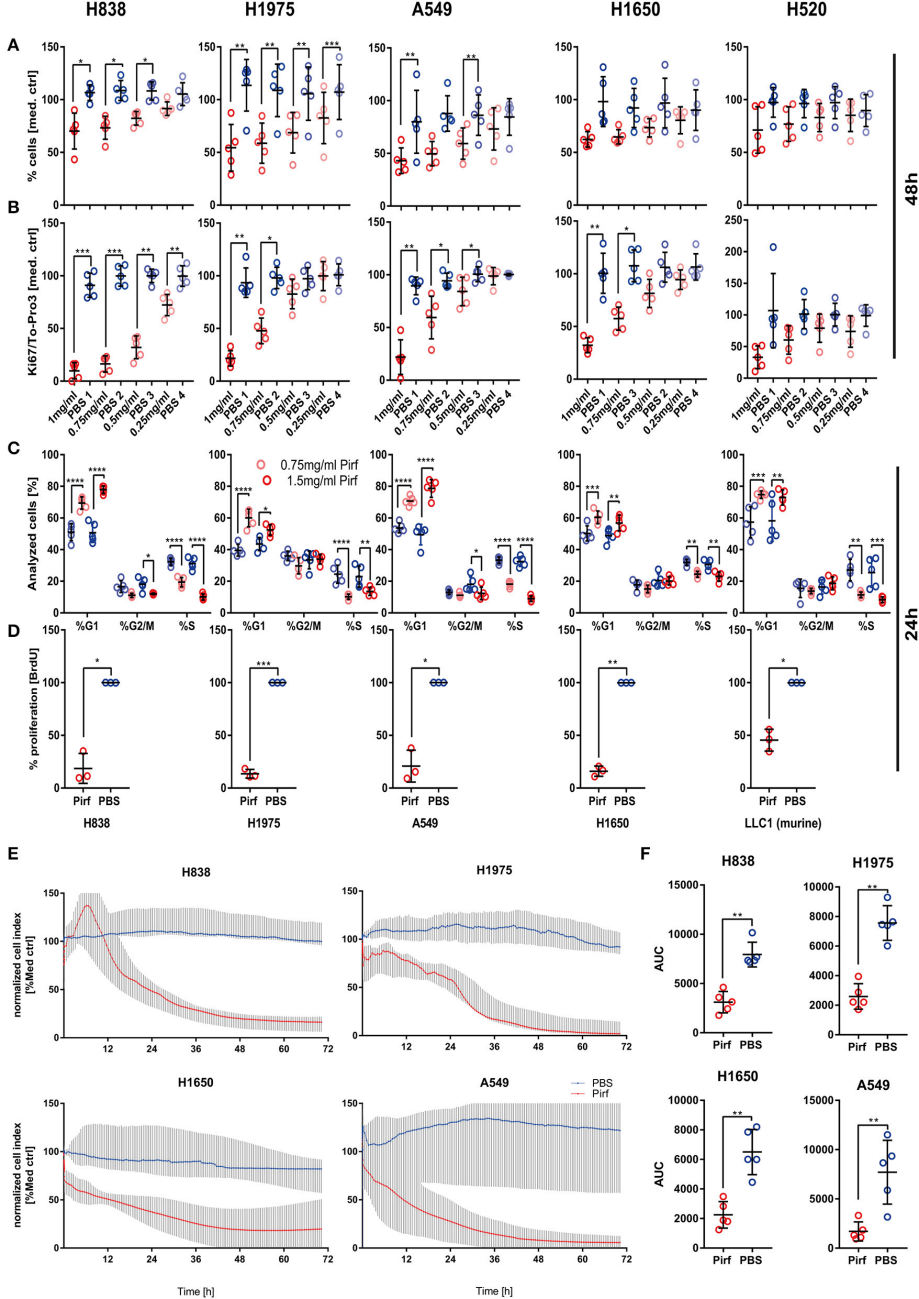
*In vitro* effect of Pirfenidone on NSCLC cell viability and proliferation. **(A,B)** Potential anti-proliferative effects and impact on cell viability were tested in four adenocarcinoma cell lines (H838, H1650, H1975, A549) and one squamous cell carcinoma cell line (H520) by relative quantification of cell number based on the To-Pro3 signal **(A)** between that in a vehicle control of PBS and multiple concentrations of Pirfenidone (1, 0.75, 0.5, 0.25 mg/ml) after 48 h of stimulation (*N* = 5). Cellular proliferation was quantified during same experiments by targeting Ki67 **(B)**. Data has been corrected for differences in cell numbers based on To-Pro3 signal and normalized to medium control. Data shown as mean ± SD with RM 1W-ANOVA and Bonferroni's multiple testing correction. **(C)** Cell cycle analysis on all cell lines was conducted after 24 h by flow cytometry and DAPI staining on Pirfenidone treated cells (0.75 and 1.5 mg/ml) and data analyzed by 2W -ANOVA with Tukey's multiple comparison test (*N* = 5). **(D)** Validation of the impact of Pirfenidone for 24 h was performed in an independent laboratory by targeting BrdU incorporation (*N* = 3) in 4 human NSCLC cell lines and 1 murine (LLC1) cell line using 1.5 mg/ml of Pirfenidone and PBS vehicle control. **(E)** Relative quantification of cell numbers over prolonged time based on impedance measurements using the Xcelligence platform was performed and data normalized to a medium control and represented as the median of *N* = 5 with 95% CI. **(F)** Area under the curve (AUC) from each experiment was calculated and Pirfenidone (1.5 mg/ml) treated cells compared with PBS treated cells by using the paired *T*-Test. For all statistical analysis *p ≤ 0.05, **p ≤ 0.01, ***p ≤ 0.001, ****p ≤ 0.0001 were considered to be indicative of statistical significance.

A growing body of data suggested an activated TGFβ signaling cascade in NSCLC (5) and recent studies showed benefits of inhibiting the TGFβ pathway (8, 9). We therefore set out to explore the impact of Pirfenidone, an approved drug for IPF treatment (14), on various human NSCLC cell lines. We first analyzed the potential anti-tumor activity by increasing concentrations of Pirfenidone on five NSCLC cell lines. Overall, a significant effect on the cell number after 48 h was observed in H838, H1975, and A549 cells (Figure 1A) which was accompanied by a significant reduction of Ki67 (Figure 1B). Further investigations were performed to analyze the effects on cell cycle. All cell lines exhibited a significant reduction of cells in the S phase and a significant increase of cells residing in the G0/G1 phase, which indicated cell cycle arrest (Figure 1C). These findings were further validated independently by using BrdU labeling to assess proliferation of four human and one murine cell line (Figure 1D). To determine if the effect of Pirfenidone exerted over a prolonged time-period, a real-time analysis of cellular viability was conducted in a time-frame up to 72 h (Figure 1E). Pirfenidone treated cells displayed a persistent reduction in the cell index over time, resulting in significant differences of Pirfenidone treated A549, H838, H1650, and H1975 cells compared to PBS treated cells (Figure 1F). Further investigation using transcriptome analysis depicting significantly regulated genes via Pirfenidone treatment for biological pathway enrichment. The evaluation resulted in a list of top 25 enriched pathways dominated almost exclusively by processes related to cell cycle, mitosis, and cell cycle checkpoints (Table 1). Overall, Pirfenidone exhibited potent efficacy on proliferation in five different NSCLC cell lines that resulted in reduced proliferation and a cell cycle arrest.

**Table 1:** Pirfenidone treatment of A549 cells leads to enriched signaling pathways involved in cell cycle, mitosis, and DNA replication.

**Pathway name**	**Source**	**Pathway ID**	**Adj. *p*-value**	**Coverage [%]**
Cell cycle	REAC	REAC:R-HSA-1640170	1.96E-13	17.56
Cell cycle, Mitotic	REAC	REAC:R-HSA-69278	2.28E-12	18.22
Retinoblastoma gene in cancer	WP	WP:WP2446	6.63E-09	33.33
Mitotic prometaphase	REAC	REAC:R-HSA-68877	7.86E-08	22.56
Resolution of sister chromatid cohesion	REAC	REAC:R-HSA-2500257	2.4956E-06	25.20
M phase	REAC	REAC:R-HSA-68886	4.9795E-06	16.62
Cell cycle checkpoints	REAC	REAC:R-HSA-69620	2.3064E-05	17.65
Cell cycle	KEGG	KEGG:04110	2.6264E-05	22.58
Spliceosome	KEGG	KEGG:03040	4.1917E-05	21.64
Mitotic anaphase	REAC	REAC:R-HSA-68882	9.0806E-05	19.49
Mitotic metaphase and anaphase	REAC	REAC:R-HSA-2555396	0.0001	19.39
Separation of sister chromatids	REAC	REAC:R-HSA-2467813	0.0003	19.25
Amplification of signal from the kinetochores	REAC	REAC:R-HSA-141424	0.0003	25.00
Amplification of signal from unattached kinetochores via a MAD2 inhibitory signal	REAC	REAC:R-HSA-141444	0.0003	25.00
Cell cycle	WP	WP:WP179	0.0003	22.50
RHO GTPases activate formins	REAC	REAC:R-HSA-5663220	0.0004	21.32
G1 to S cell cycle control	WP	WP:WP45	0.0006	28.13
Mitotic spindle checkpoint	REAC	REAC:R-HSA-69618	0.0015	22.22
TP53 regulates transcription of cell cycle genes	REAC	REAC:R-HSA-6791312	0.0017	30.61
G1/S transition	REAC	REAC:R-HSA-69206	0.0018	20.61
Mitotic G1-G1/S phases	REAC	REAC:R-HSA-453279	0.0023	19.59
Processing of capped intron-containing pre-mRNA	REAC	REAC:R-HSA-72203	0.0023	16.81
Telomere C-strand (lagging strand) synthesis	REAC	REAC:R-HSA-174417	0.0033	41.67
S phase	REAC	REAC:R-HSA-69242	0.0045	18.63
DNA replication	KEGG	KEGG:03030	0.0072	30.56

### Pirfenidone Significantly Affected Core TGFβ Signaling Mediators and C-Terminal Phosphorylation

**Figure 2:**
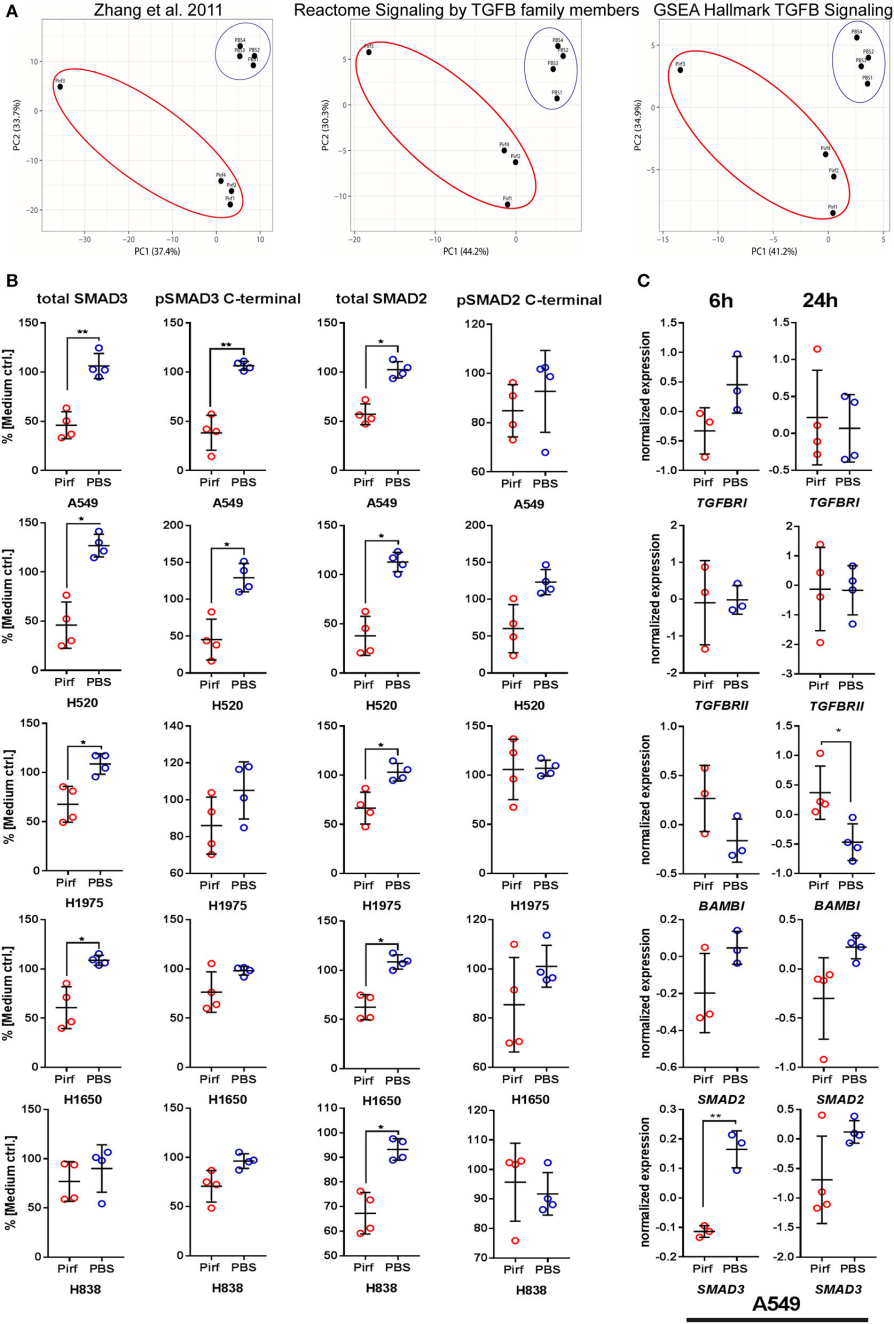
Pirfenidone influences TGFβ pathway member expression and regulation on mRNA and protein level. **(A)** mRNA expression from transcriptome data of Pirfenidone or PBS treated A549 cells after 24 h. Principal Component Analysis (PCA) of gene sets targeting TGFβ 1/SMAD3-downstream genes in A549 cells (19) or Reactome and GSEA Hallmark gene sets. **(B)** In-Cell Western assay targeting relative expression of total SMAD2, total SMAD3, and C-terminal phosphorylation of SMAD2/-3 normalized to total amount of cells and expression in medium controls. **(C)** Transcriptome data of selected pathway mediators from 6 to 24 h treatment of A549 cells with 1.5 mg/ml Pirfenidone. Shown as mean values (*N* = 3–4) ± SD. Paired *T*-Test with *p ≤ 0.05, **p ≤ 0.01, were considered to be indicative of statistical significance.

The exact molecular mechanism of Pirfenidone has not yet been totally elucidated, but effects on the TGFβ pathway downstream targets TGFβ 1, Collagen I, and HSP47 have been discussed (15, 18). To investigate if Pirfenidone exerts a direct effect on the signaling cascade, we first analyzed the transcriptome of A549 cells and the response of three specific TGFβ pathway gene sets. PCA analysis demonstrated a clear distinction of A549 cells treated with Pirfenidone vs. A549 cells treated with PBS. This was true in the GSEA “Hallmark TGFβ” gene set, Reactome “Signaling by TGFβ family members” gene set as well as the gene set from Zhang et al. (19). This gene set in particular was derived from ChIP experiments of TGFβ stimulated A549 cells and resulted in a set of genes which were shown to be regulated by SMAD3 binding to their promotor region (Figure 2A). We next aimed to analyze the effect on TGFβ core pathway mediators and their biological activities on protein level to assess possible biological relevance related to SMAD expression and phosphorylation. Both SMAD2 and SMAD3 were investigated for their relative protein abundance and their C-terminal phosphorylation. Treatment of NSCLC cell lines with Pirfenidone caused significant reduction in total SMAD3 in the A549, H1650, H1975, and H520 cell lines and a similar trend in H838 (Figure 2B) were observed. Additionally, the C-terminal phosphorylation of SMAD3 was significantly reduced in A549 and H520 cell lines with a similar trend for H838, H1650, and H1975. Similarly, a significant reduction of total SMAD2 protein was observed in all analyzed cell lines with no significant reduction in phosphorylation of SMAD2, although decreasing trends were observed in A549, H1650, and H520 cells. A matching pattern was detected on RNA level, where *SMAD3* was significantly reduced upon Pirfenidone stimulation after 6 h of treatment with a concomitant increase of the TGFβ pseudo-receptor *BAMBI* after 24 h (Figure 2C).

### Pirfenidone Reduced the Tumor Growth and Improved Tumor Infiltration by Immune Cells

**Figure 3:**
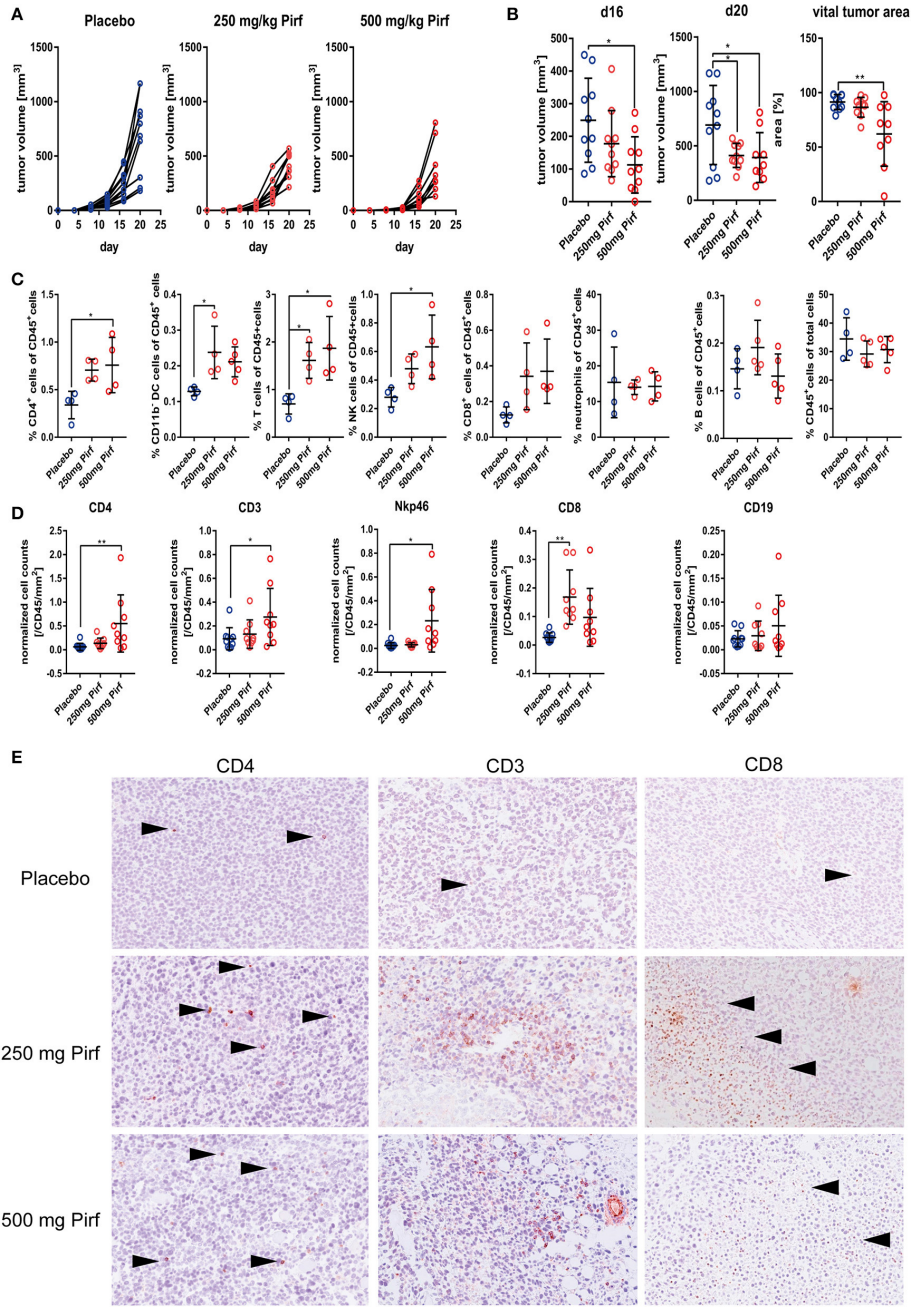
Effect of Pirfenidone in subcutaneously transplanted LLC1 cells. **(A)** Measurement of tumor volume during course of *in vivo* experiment with 250 mg/kg body weight Pirfenidone, 500 mg/kg body weight or respective vehicle control (Placebo). **(B)** Quantification of tumor volume at d16 and d20 as well as percentage of vital tumor area at d20 comparing Pirfenidone treated groups with Placebo control group. **(C)** Flow cytometry analysis of digested tumor tissues for relative abundance of invaded immune cell populations targeting CD45+ cells, CD4+, CD8+, neutrophils, B cells, CD11b– DCs, T cells, and NK cells. Statistical evaluation performed as indicated above. **(D)** Additional phenotyping of intra-tumoral immune cells as performed by IHC targeting CD4, CD8, CD19, CD3, and Nkp46 with exemplary images of CD3, CD4, and CD8 IHC taken at 20x magnification **(E)**. 1W-ANOVA with Dunnett's multiple comparison test considering *p ≤ 0.05, **p ≤ 0.01 significant was used for **(B–D)**.

Since we observed potent effects of Pirfenidone on NSCLC cell proliferation and viability as well as an impact on TGFβ pathway, we next investigated the effect in an *in vivo* model using the murine adenocarcinoma cell line LLC1 as recent publications highlighted the possible benefits of interfering with the TGFβ pathway to support radiation therapy (20) or checkpoint therapy (8, 9, 21, 22). We choose two different dosages of Pirfenidone based on previous studies in animal models of fibrotic diseases (23–25). Pirfenidone slowed down the tumor growth, as indicated by tumor volume over time (Figure 3A) with a significant reduction at day 16 at the highest dose of Pirfenidone and at both concentrations by the end of the experiment (Figure 3B). The vital tumor area was significantly reduced in mice that were treated with 500 mg/kg of Pirfenidone (Figure 3B). At day 20, some tumors were harvested and investigated by flow cytometry for the proportion of infiltrated immune cells. The overall number of CD45^+^ cells did not change significantly, but the percentage of total T cells as well as CD4^+^ helper T cells, NK cells, and CD11b^−^ dendritic cells exhibited a significant increase (Figure 3C). The percentage of CD8^+^ cytotoxic T cells showed a clear tendency that did not reach statistical significance, whereas the abundance of neutrophils seems to be unaffected by both concentrations of Pirfenidone. Paraffin-embedded tumor tissues were then investigated histologically for their vital tumor area and immune cell markers by IHC (Figure 3D). As observed by flow cytometry, the amount of CD4^+^ cells increased significantly as well as CD3^+^ (Figures 3D,E) and Nkp46^+^ cells with Pirfenidone treatment within the tumor tissues, whereas CD19^+^ cells were unaffected by the treatment. Thus, Pirfenidone treatment reduced tumor growth in a murine mouse model and was accompanied by reduced vital tumor area and a significant increase in immune cells, primarily in T cells and NK cells.

### Pirfenidone Down-Regulated Survivin, Affected Apoptotic Signaling and Mitigated Migratory as Well as Wound Healing Capacity

**Figure 4:**
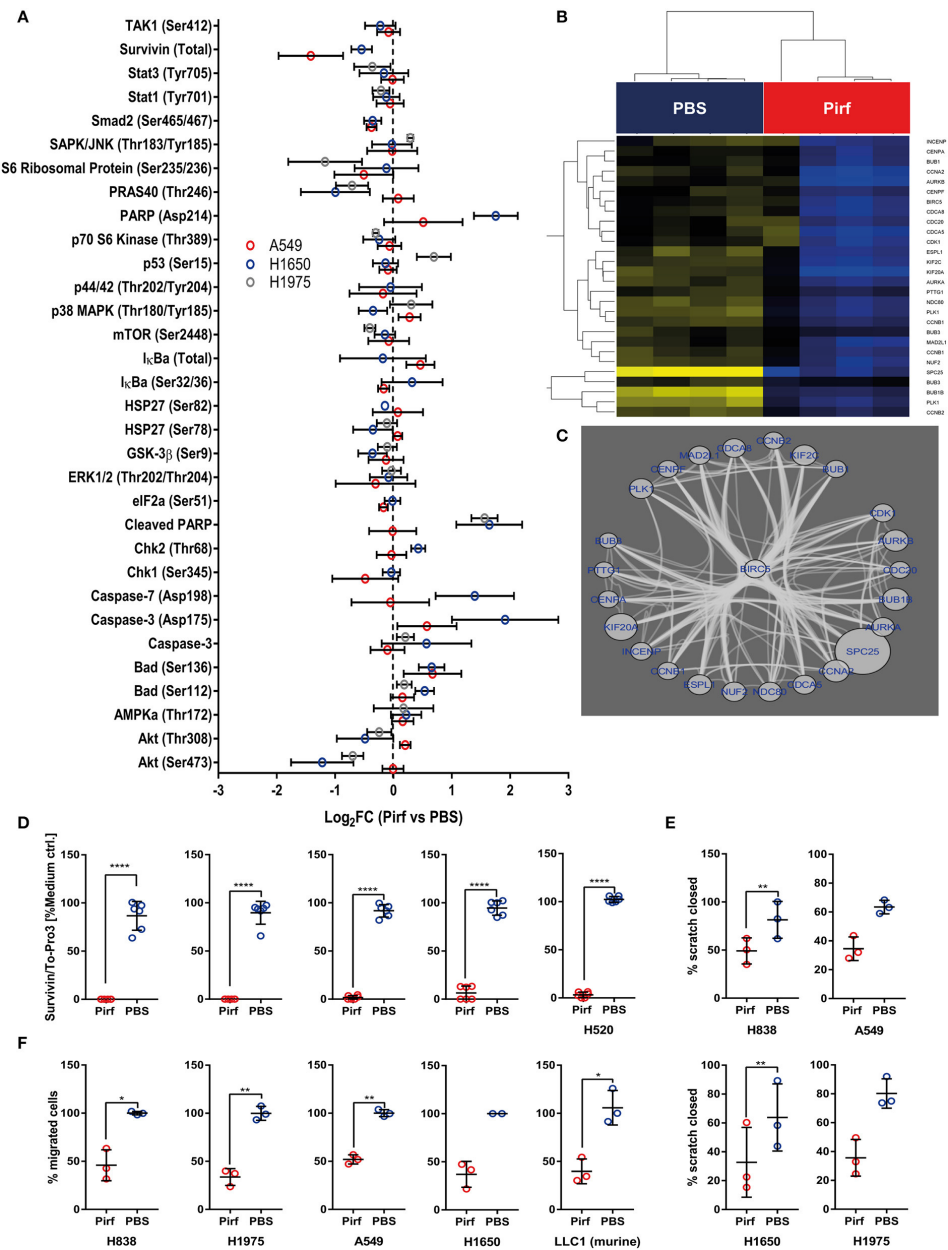
Pirfenidone regulates cell stress mediators and negatively impacts pro-survival molecule Survivin. **(A)** Effect of Pirfenidone (1.5 mg/ml) or a respective volume of PBS on cell stress and different intracellular signaling nodes after 24 h. Antibody array analyses of lysates from cell culture experiments. Seventy-five micrograms amount of total protein was hybridized to Apoptosis and Cell Stress Arrays and Intracellular Signaling Arrays. Relative fluorescence signal of each experimental parameter was normalized to respective medium control and mean values of technical replicates from each arrays used to compute the Median with 95% CI from biological replicates: Cell stress array A549 (*N* = 4) and H1650 (*N* = 2–4) or Intracellular signaling array A549 (*N* = 3), H1975 (*N* = 3), and H1650 (*N* = 4). Data displayed as Log_2_ Fold Changes of Pirfenidone treated cells in comparison to PBS control. **(B)** BIRC5 regulatory network as enriched by STRING protein-interaction database was analyzed for significantly expressed genes on RNA level between PBS and Pirfenidone treated A549 cells. **(C)** Significantly regulated genes by Pirfenidone within the Survivin/BIRC5 network of interacting molecules display high level of connectivity. Down-regulation by Pirfenidone indicated by blue font and bubble size corresponds to the magnitude of fold change. **(D)** Effect of Pirfenidone (1.5 mg/ml) on the protein expression of Survivin after 24 h. In-Cell Western assay targeting relative expression of Survivin, normalized to the total amount of cells and expression in medium controls (*N* = 4). **(E)** Scratch assay of Pirfenidone treated cells (1.5 mg/ml) or PBS control, Data displayed as % of closed scratch area comparing initial area and 20 h post wounding (*N* = 3). **(F)** Migration assay after 16 h treatment of Pirfenidone (1.5 mg/ml) or PBS. Data displayed as the percentage of migrated cells to PBS control or scratched closed (*N* = 3). All data shown as mean ± SD. Statistical analysis for **(D–F)** by Paired *T*-Test with *p ≤ 0.05, **p≤0.01, ****p ≤ 0.0001.

As the *in vivo* data suggested a reduction in vital tumor area that was accompanied by an increased influx of inflammatory cells, we speculated if Pirfenidone might exert effects on cellular health in addition to the observed effects on proliferation. Therefore, we assessed effects on non-TGFβ pathway related mediators by analyzing lysates from Pirfenidone treated NSCLC cell lines for cell stress markers along with other intracellular signaling events (Figure 4A). In A549 cells, a reduction in Survivin expression was observed as well as a distinct trend in the H1650 cell line (Figure 4A). Survivin is thought to prevent apoptosis and induce cell proliferation in multiple malignancies (26) and conveys prognostic value for stratifying lymph-node positive NSCLC patients (27). We further validated the antibody array findings by ICW assay, which resulted in significant reduction of Survivin expression among all investigated NSCLC cell lines (Figure 4D). In addition we queried the String protein-protein interaction database for other molecules that are likely to biologically interact with Survivin (Figure 4C). These molecules were linked back to the transcriptome data and investigated for significant regulation by Pirfenidone. Here, all of the mutual interactors of Survivin exhibited significant regulation, which led to a reduced expression of these molecules in Pirfenidone treated A549 cells (Figure 4B). Furthermore, Survivin/BIRC5 interacted closely with molecules (Figure 4C) which were shown to regulate stem cell properties in lung adenocarcinomas (SPC25) (28), influence radiosensitivity in lung cancer (AURKA) (29), or mediate anti-EGFR therapy in NSCLC (AURKB) (30). Interestingly, a majority of Survivin interacting molecules that were found to be significantly regulated by Pirfenidone are also associated with the mitotic spindle (MAD2L1, BUB1, BUB1B, BUB3, CDC20) and were shown to be increased in human breast cancer (31). Furthermore, phosphorylated Chk1 seemed to be reduced upon Pirfenidone treatment. Chk1 is involved in DNA damage repair and necessary for checkpoint delay (32), thereby complementing the cell cycle results. Expression of pro-apoptotic Caspase-3/-7 and phosphorylation as well as cleavage of PARP was up-regulated in H1650 or H1975 (Figure 4A) but not A549 cells which might be due to differences in the genetic background of the analyzed cell lines. Finally, we characterized the effect of Pirfenidone on the wound healing and migratory capacity of 4 human and 1 murine adenocarcinoma cell line (Figures 4E,F). Here, Pirfenidone significantly reduced the wound healing capabilities in H838 and H1650 cells with a similar trend observed in A549 and H1975 cells (Figure 4E). Moreover, migration was successfully reduced in all investigated cell lines by treatment with Pirfenidone (Figure 4F). Taken together, Pirfenidone affected several proteins that are involved in cell cycle regulation, stress response, DNA damage repair, or exhibit prognostic information on response to different therapy regimen. The impact on these molecules was accompanied by reduced migration and wound healing capacity thereby indicating a decreased metastatic potential.

## Discussion

In this study, we investigated the effect of the single-drug potency of Pirfenidone on NSCLC cell lines and an *in vivo* tumor model. In general, Pirfenidone exerted an effect on cellular proliferation, as indicated by reduced Ki67 protein level and BrdU incorporation, as well as G0/G1 arrest in the cell cycle in five different NSCLC cell lines. Furthermore, core mediators of the signaling pathway showed effects on their expression at the mRNA and the protein level, where Pirfenidone induced a differential gene expression response in three well characterized gene sets comprising SMAD3 downstream genes (19) or TGFβ pathway members. A similar effect on cell proliferation was recently observed in mesothelioma cell lines (33), hepatocellular carcinoma cells (34), and pancreatic cancer cells (16), although there was no effect on SMAD expression or phosphorylation but rather on p-ERK 1/2 (33) in mesothelioma cells or p-Stat3 in fibroblasts of IPF patients (35). Pirfenidone affected also cellular mobility as indicated by reduced wound healing ability and migration that was also recently observed independently and linked to the uPA/PAI-1 system (17) and resulted in our experiments in a reduced vital tumor area *in vivo*. As Pirfenidone not only regulated TGFβ pathway members but also other molecules such as Survivin/BIRC5 among others, we cannot exclude the possibility that the observed effects are not directly TGFβ -dependent or TGFβ -exclusive. Nevertheless, Pirfenidone might target other pathways, as it was shown to influence pro-malignant processes and molecules such as the pro-survival molecule Survivin (26, 27) or PRAS40, a negative regulator of the p53 nuclear stress response pathway and promoter of cell survival and tumorigenesis (36) in addition to pro-apoptotic molecules PARP and cleaved Caspase 3/7 (Figure 4A). Regarding induction of apoptosis and differentiation from autophagy or senescence, we assume that the mutational background of the investigated cells might be discriminating factor since A549, H1650, or H1975 cells responded slightly different to Pirfenidone.

Nevertheless, the overall results among all investigated cell lines clearly pointed out the potent anti-tumor activity and a positive effect toward TME reprogramming, as Pirfenidone successfully enhanced recruitment of immune cells into the tumor. Furthermore, by altering the immune cell composition in the TME toward an induction of NK cells or T cells, Pirfenidone may act also on different layers: A direct anti-tumor effect targeting the proliferative potential of NSCLC cells and inducing pro-apoptotic molecules and an indirect anti-tumor effect by attracting NK and T cells.

Recent observations showed promising evidence of clinical efficacy in malignant diseases for inhibiting TGFβ alone or together with PD-L1 as it was shown that response to PD-L1 therapy in metastatic urothelial cancer was attenuated by a TGFβ gene expression signature in fibroblasts contributing to exclusion of CD8 cells. Recapitulation in a mouse model targeting TGFβ and PD-L1 together, lead to improved T cell invasion into the tumor, anti-tumor immunity and tumor regression (21). Also application of the bifunctional fusion protein M7824, which targets PDL1 and TGFβ 1 (10). Pirfenidone has been shown to synergize with cisplatin in killing tumor cells and CAFs in NSCLC cells (37) as well as to act synergistically with doxorubicin in a triple-negative breast cancer (TNBC) model and inhibiting tumor fibrosis and TGFβ signaling (38); although individual effects of Pirfenidone on tumor growth and lung metastases were not evaluated so far. Again, an effect on TME normalization has been previously observed that affected ECM molecules and blood vessel functionality and resulted in improved doxorubicin efficacy based on improved drug delivery in two orthotopic murine breast cancer models (11).

We therefore aim to highlight the individual efficacy of Pirfenidone in inhibiting TGFβ pathway activation with subsequent recruitment of effector immune cells that result in diminished tumor growth and tumor size *in vivo*, which still leaves open mechanistic questions on the exact molecular mode of action. Nevertheless, we perceive a solid benefit of using an FDA/EMA approved drug repurposed for application in NSCLC therapy that leaves potential for further investigations with regards to combinations with checkpoint therapy to improve immune cell activation and recruitment concomitantly with anti-tumorigenic effects on cell cycle and central stress-related pathways induced within the tumor.

## Data Availability Statement

Publicly available datasets were analyzed in this study, these can be found in the NCBI Gene Expression Omnibus (GSE136935).

## Ethics Statement

All experiments using animal models were performed according to the German Law for Animal Protection and the National Institute of Health Guidelines for Care and Use of Laboratory Animals. The protocol for the animal studies was approved by the University Animal Care Committee and the federal authorities for animal research of the Regierungspräsidium Darmstadt (Hessen, Germany; approval number B2/1107).

## Author Contributions

TG, RS, KT, and SM conceived of the study, planned the experiments, and drafted the manuscript. SM, KT, DN, JB, and AW conducted experiments, analyzed data, and produced figures. MR, NR, MT, KR, WS, and DD provided critical (clinical) insights. All authors read and approved the final manuscript.

### Conflict of Interest

The authors declare that the research was conducted in the absence of any commercial or financial relationships that could be construed as a potential conflict of interest.
